# Short-term outcome of totally laparoscopic gastrectomy for gastric cancer: a comparative study

**DOI:** 10.1016/j.clinsp.2026.100887

**Published:** 2026-02-19

**Authors:** Liu Liu, Shijie Feng, Haiyan Wang, Lin Liu

**Affiliations:** a Department of General Surgery, The First Affiliated Hospital of USTC, Division of Life Sciences and Medicine, University of Science and Technology of China, China; b Department of General Surgery, The Anhui Provincial Hospital affiliated to the Anhui Medical University, China; c Department of General Surgery, Anhui Provincial Hospital, Bengbu Medical University, China; d Department of Anesthesiology, The First Affiliated Hospital of USTC, Division of Life Sciences and Medicine, University of Science and Technology of China, China

**Keywords:** Gastric cancer, Laparoscopic surgery, Gastrectomy, Surgical recovery, Complication

## Abstract

**Objective:**

Whether Totally Laparoscopic Gastrectomy (TLG) is beneficial for patients with gastric cancer remains not fully understood. This study was to evaluate the advantages of Totally Laparoscopic Gastrectomy (TLG) for treating Gastric Cancer (GC).

**Methods:**

From January 2019 to December 2021, 380 patients with gastric cancer were included into this study (190 per group). The outcomes of interest, including postoperative recovery, inflammation, and oncological results, were compared between the two groups.

**Results:**

The patient baselines between the two groups were comparable. The TLG group had lower risks of wound infection (*p* = 0.02) and total complications (*p* = 0.03) compared to LAG. The TLG group exhibited significantly shorter operative time (median: 215 vs. 240 min, *p* < 0.001), less intraoperative blood loss (median: 50 vs. 100 mL, *p* < 0.001), shorter time to first flatus (median: 3 vs. 4 days, *p* < 0.001), and reduced length of postoperative hospital stay (*p* < 0.001). Furthermore, patients in the TLG group exhibited lower White Blood Cell (WBC) counts on POD 1 compared to patients in the LAG group (mean 11.74 vs. 12.75 × 10^9/L; *p* = 0.003). Multivariate logistic regression analysis revealed that TLG was an independent predictor of shorter postoperative hospitalization (OR (95% CI): 0.54 (0.30‒0.95), *p* = 0.03). Interestingly, the TLG group had more lymph nodes harvested than the LAG group (median (range): 23 (2‒88) vs. 23 (2‒88), *p* < 0.001).

**Conclusions:**

This study demonstrates that TLG has a lower risk of complications and faster postoperative recovery, thus TLG is superior to LAG for the treatment of gastric cancer.

## Highlights

• Totally Laparoscopic Gastrectomy (TLG) has been used for the treatment of gastric cancer.
• Compared with Laparoscopy-Assisted Gastrectomy (LAG), TLG has a shorter operative time and less intraoperative blood loss.
• TLG contributes to faster recovery and shorter time of hospitalization than LAG.

## Introduction

Compared to open gastrectomy, laparoscopic radical gastrectomy for patients with gastric cancer features a smaller incision size, which not only reduces intraoperative injury but also achieves a successful reduction in postoperative recovery time.^1^, ^2^, ^3^, ^4^ Randomized controlled trials, such as CLASS-01, CLASS-02, and KLASS-02, have demonstrated that patients undergoing laparoscopic-assisted gastric surgery experience reduced risk of complications and faster recovery compared to those undergoing open surgery.^2^^,^^5^^,^^6^ As a result, laparoscopic radical gastrectomy is increasingly favored as a surgical option for gastric cancer patients worldwide.

Laparoscopic gastric resection encompasses two specific techniques: Totally Laparoscopic Gastric Resection (TLG) and Laparoscopic-Assisted Gastric Resection (LAG). In TLG, all procedures, including gastric resection, lymph node dissection, and gastrointestinal reconstruction, are performed within the abdominal cavity. Conversely, in LAG, the reconstruction is carried out outside the abdominal cavity through a 10 cm abdominal incision. Compared to LAG, TLG presents greater technical complexity for surgeons due to some technical challenges: the confined abdominal field during gastrointestinal reconstruction; the difficulty in manipulating a linear stapler during anastomosis; and the time-consuming nature of anastomotic reinforcement. Over the past decade, advancements in laparoscopic equipment and improvements in surgeons' laparoscopic skills have significantly increased the interest in TLG as a preferred option for the treatment of gastric cancer.^7^

Although Totally Laparoscopic Distal Gastrectomy (TLDG) has been performed and offers theoretical advantages in promoting postoperative recovery, its benefits have not been fully demonstrated yet. Previous studies have compared the postoperative outcome between TLDG and Laparoscopy-Assisted Distal Gastrectomy (LADG),^3^^,^^5^^,^^8^, ^9^, ^10^, ^11^, ^12^ however, inconsistent findings have been reported, warranting further investigation. Some studies^3^^,^^5^^,^^8^^,^^9^^,^^11^ have reported that TLDG significantly shortens hospital stay, enhances postoperative recovery, and does not increase the risk of postoperative morbidities compared to LADG. However, other studies^10^^,^^12^ have demonstrated that TLDG does not offer any superiority over LADG in terms of postoperative inflammation, recovery, and complication risk. Similarly, inconsistent findings have been reported in other studies comparing Laparoscopic Assisted Total Gastrectomy (LATG) and Totally Laparoscopic Total Gastrectomy (TLTG).^13^, ^14^, ^15^ Considering inconsistent results reported, this study aimed to evaluate the 30-day postoperative outcomes of Total Laparoscopic Gastrectomy (TLG) versus Laparoscopic Assisted Gastrectomy (LAG) in a total of 380 patients (190 patients in each group) with gastric cancer.

## Materials and methods

### Study design

**Fig. 1:**
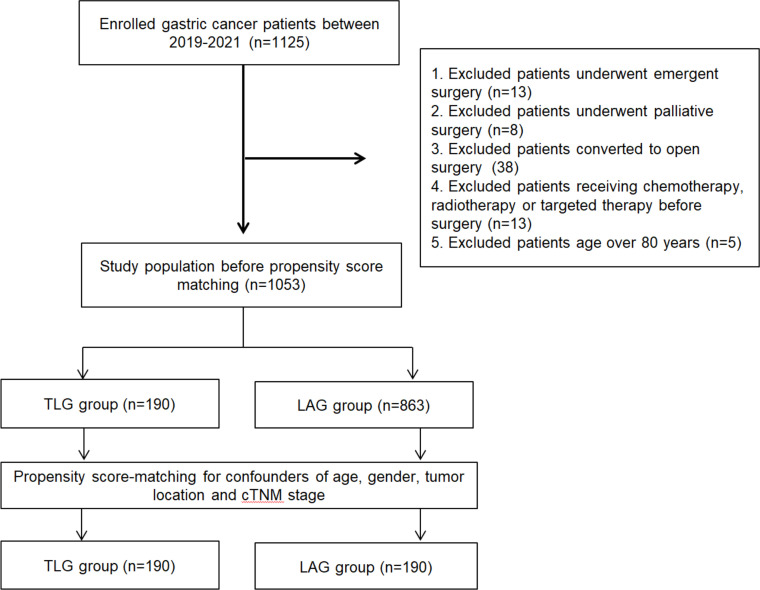
The flow chart for patient selection.

This retrospective cohort study was conducted according to the STROBE statement.^16^ A total of 1125 patients with gastric cancer were identified and screened. From these patients, 190 patients undergoing TLG between January 2019 and December 2021 at the studied department were included (TLG group). Meanwhile, 863 eligible patients undergoing LAG during the same period were identified, and 190 patients were selected as the control group (LAG group) using propensity score-matching to match for age, sex, tumor location and cTNM, as previously described.^17^ Fig. 1 was the flowchart for patient selection. Before surgery, all patients provided written informed consent. This study was approved by the Ethics Committee of the First Affiliated Hospital of the University of Science and Technology of China (n° 2022-RE-216) and registered on https://www.chictr.org.cn (register number: ChiCTR2200064119).

### Study population

The eligibility criteria for inclusion were as follows: 1) Gastric adenocarcinoma diagnosed by endoscopic biopsy; 2) Tumors located at the antrum, horn, or body of the stomach. Regarding to the proximal third gastric cancer, Siewert II esophagogastric junction adenocarcinoma and Siewert III adenocarcinoma (proximal gastric tumor) were included; 3) Age range: 18 to 80-years-old; 4) ASA physical status I, II, or III; 5) Ability to provide informed consent for TLG or LAG surgery; 6) Curative surgical resection performed.

Exclusion criteria were as follows: 1) Emergent gastrectomy due to massive bleeding, perforation, or outlet obstruction; 2) Palliative or conversion to palliative gastrectomy; 3) Combined organ resection (excluding cholecystectomy); 4) History of upper abdominal surgery (excluding cholecystectomy); 5) History of prior systemic inflammatory disease; 6) Current receipt of chemotherapy, radiotherapy, or targeted therapy; 7) Presence of severe cardiovascular or cerebrovascular disease within the past 6-months; 8) Cannot provide informed consent, pregnancy, or lactation status.

### Surgical procedure

Laparoscopic gastric resection and lymphadenectomy were performed in accordance with the Japanese Gastric Cancer Treatment Guidelines 2014.^18^ D1-pus lymphadenectomy or D2 lymphadenectomy was conducted for early gastric cancer (T1a or T1b) or locally advanced gastric cancer. On the morning of surgery, the tumor lesion was marked by an endoscopic clip and methylene blue to facilitate visualization of early gastric cancer.

In the TLG group, the digestive tract anastomosis was performed in the abdominal cavity, whereas in the LAG group, the anastomosis was conducted outside the abdominal cavity. The Billroth-II-Braun anastomosis was used in patients who underwent distal gastrectomy, while the Roux-en-Y reconstruction was employed for total gastrectomy patients. In the Roux-en-Y procedure, the esophagus was transected, and the jejunum was transected 20 cm below the Treitz ligament using a linear stapler. A side-to-side esophagojejunostomy was performed using the esophageal and jejunum stumps. Subsequently, a jejunojejunostomy was conducted 45 cm below the esophagojejunal anastomosis. An upper abdomen incision of 3‒5 cm was made for sample removal. All surgeries were performed by experienced surgeons with >50 cases of laparoscopic gastric surgery each year.

### Data collection and outcome

The data collected included the following patient characteristics: sex age, body mass index (BMI), Charlson Comorbidity Index (CCI), comorbidities according the American Society of Anesthesiologists (ASA) score, and results of blood tests, which included White Blood Cell (WBC) count, Neutrophil-to-Lymphocyte Ratio (NLR), and lymphocyte count. Additionally, postoperative data were recorded, including operative time, Estimated Blood Loss (EBL), time to first flatus, time to first liquid diet, length of postoperative hospital stay, and postoperative complications. Postoperative complications were defined according to the Clavien-Dindo classification,^19^ with major complications classified as Clavien-Dindo Grades 3, 4, or 5. Postoperative inflammation was assessed using WBC count, NLR, and serum Albumin (ALB) levels on postoperative days-1 and −3. Oncologic outcomes included tumor size, location, TNM stage, tumor differentiation, proximal or distal margins, and the number of lymph nodes harvested. Pathological staging of gastric cancer was performed according to the TNM staging system of the American Joint Committee on Cancer (AJCC) 8th edition.

### Statistical analysis

Continuous data were expressed as mean ± Standard Deviation (SD) if normally distributed; otherwise, the median (interquartile) was used. Continuous data were compared using Student’s *t*-test for normally distributed data or the Mann-Whitney *U* test for non-normally distributed data. Categorical variables were analyzed using the Chi-Squared test or Fisher's exact test, depending on whether the cell count exceeded 5. The length of postoperative hospitalization duration (continuous data) was dichotomized at the 75th percentile (9-days) to create a binary variable. Univariate and multivariate logistic regression analyses were performed to identify independent predictors of postoperative hospital stay duration and complications. Statistical analyses were conducted using SPSS (version 26.0; IBM Corporation, Armonk, NY, USA).

## Results

### Patient characteristics

From January 2019 to December 2021, 190 patients undergoing TLG were included in this study. Among these patients, 76 patients underwent TLTG and 114 underwent TLDG. A total of 190 patients undergoing LAG were selected as the control group (Fig. 1). Table 1 shows the baseline of patients in the two groups. In the TLG group, 73 % (138/187) of the patients were male, with a mean (SD) age of 61.2 ± 10.5 years. The mean (SD) age of patients in the LAG group was 61.6 ± 10.6 years. Other baseline characteristics, including Body Mass Index (BMI), Clinical Classification Index (CCI), history of abdominal surgery, preoperative white blood cell count, Hemoglobin (Hb) concentration, and Albumin (ALB) level, were comparable between the two groups. Subgroup analyses of baselines for patients receiving total gastrectomy or distal gastrectomy were also comparable between the totally laparoscopy group and the laparoscopy-assisted group (Supplementary Table 1).

**Table 1:** The baseline characteristics of patients.

Variables	TLG group (*n* = 190)	LAG group (*n* = 190)	*p*-value
**Age (years), (mean ± SD)**	61.2 ± 10.5	61.6 ± 10.6	0.69
**Sex**			0.73
Male	138	135	
Female	51	55	
**BMI (kg/m^2^), (mean ± SD)**	23.0 ± 2.9	22.6 ± 2.9	0.21
**Charlson Comorbidity Index (CCI)**			0.35
0‒1	177	172	
> 1	13	18	
**ASA score**			0.14
I & II	64	78	
III	126	112	
**History of abdominal surgery**			0.26
Yes	18	25	
No	172	165	
**Tumor location**			0.36
Upper	42	45	
Middle	42	31	
Lower	106	114	
**Gastrectomy**			1.00
Total gastrectomy	76	76	
Distal gastrectomy	114	114	
Preoperative WBC (× 10^9^/L), (mean ± SD)	6.0 ± 1.5	5.7 ± 1.7	0.16
Preoperative NLR, (mean ± SD)	2.7 ± 1.6	2.4 ± 1.4	0.08
Preoperative Hb (g/L), (mean ± SD)	124±23	122±20	0.40
Preoperative ALB (g/L), (mean ± SD)	40.1 ± 5.0	39.8 ± 5.0	0.67

SD, Standard Deviation; BMI, Body Mass Index; WBC, White Blood Cell; NLR, Neutrophil to Lymphocyte Ratio; Hb, Haemoglobin; ALB, Albumin; TLG, Totally Laparoscopic Gastrectomy; LAG, Laparoscopy-Assisted Gastrectomy.

### Postoperative recovery and inflammation

Postoperative recovery data are summarized in Table 2. All patients underwent an R0 resection with either D1-plus or D2 lymph node dissection. In this study, 152 patients underwent total gastrectomy with Roux-en-Y digestive tract reconstruction, while 228 patients underwent distal gastrectomy with Billroth-II reconstruction. Notably, no patients underwent combined organ dissection.

**Table 2:** Surgical outcomes and postoperative recovery.

Variables	TLG group	LAG group	*p*-value
	(*n* = 190)	(*n* = 190)	
Operative time (min), median (IQR)	215 (194‒250)	240 (210‒280)	<0.001
Estimated blood loss (mL), median (IQR)	50 (50‒100)	100 (60‒150)	<0.001
First flatus (days), median (IQR)	3 (3‒3.8)	4 (4‒5)	<0.001
Liquid diet (days), median (rang)	4 (3‒4)	6 (5‒6)	<0.001
Postoperative hospital stay (days), median (IQR)	8 (7‒9)	8 (7‒10)	<0.001
WBC (× 10^^9^/L), mean (SD)			
POD1	11.74 ± 3.10	12.75 ± 3.56	0.003
POD3	8.03 ± 2.30	7.73 ± 2.78	0.26
NLR, mean (SD)			
POD1	15.16 ± 17.46	18.31 ± 17.16	0.08
POD3	7.38 ± 6.18	8.00 ± 6.52	0.34
ALB (g/L), mean (SD)			
POD1	34.48 ± 4.36	35.16 ± 3.90	0.11
POD3	34.73 ± 4.65	35.57 ± 4.22	0.07
Medical cost (CNY), median (IQR)	55,411 (49,229‒60,507)	45,534 (41,021‒50,277)	<0.001

IQR, Interquartile; SD, Standard Deviation; WBC, White Blood Cell; NLR, Neutrophil to Lymphocyte Ratio; ALB, Albumin; TLG, Totally Laparoscopic Gastrectomy; LAG, Laparoscopy-Assisted Gastrectomy.

The TLG group exhibited significantly shorter operative times (median (IQR): 215 (194‒250) min vs. 240 (210‒280) min; *p* < 0.001), lower estimated blood loss (median (IQR): 50 (50‒100) mL vs. 100 (60‒150) mL; *p* < 0.001), and shorter times to first flatus (median (IQR): 3 (3‒3.8) days vs. 4 (4‒5) days; *p* < 0.001) and first liquid diet (median (IQR): 4 (3‒4) days vs. 6 (5‒6) days; *p* < 0.001), compared to LAG. Additionally, the TLG group demonstrated a shorter postoperative hospital stay (median (IQR): 8 (7‒9) days vs. 8 (7‒10) days; *p* < 0.001). Furthermore, compared to the LAG group, the TLG group exhibited a lower WBC count on day 1 after the operation. However, the TLG group experienced significantly higher medical costs (median (IQR): 55,411 (49,229‒60,507) CNY versus 45,534 (41,021‒50,277) CNY; *p* < 0.001).

The length of postoperative hospitalization is an important clinical outcome following a gastrectomy procedure. Multivariate logistic regression analysis was employed to evaluate the independent risk factors associated with prolonged postoperative hospitalization. The univariate analysis identified several risk factors, including LAG (but not TLG), longer operative time, increased intraoperative blood loss (intraoperative EBL), low preoperative Hemoglobin (Hb) level, low preoperative Albumin (ALB) level, and the presence of postoperative complications. Among these, the multivariate analysis revealed that LAG and postoperative complications emerged as the independent predictors of prolonged hospital stay (Table 3).

**Table 3:** Univariable and multivariable logistic regression analysis for the factors associated with prolonged hospitalization.

Variables	Univariate analysis for postoperative hospitalization			Multivariate analysis	
	Length (> 9-days), (*n* = 81)	Length (≤ 9-days), (*n* = 299)	*p*-value	Odds Ratio (95 % CI)	*p*-value
Age (years), mean (SD)	60.8 ± 10.6	63.4 ± 10.2	0.04	1.00 (0.98‒1.03)	0.80
Sex (female vs. male)	28	78	0.27	1.52 (0.82‒2.71)	0.16
BMI (kg/m^2^), mean (SD)	23.0 ± 2.9	22.8 ± 2.9	0.58		
ASA (score 3 vs. score 1 & 2)	44	180	0.62		
Total gastrectomy (yes vs. no)	42	110	0.12	1.68 (0.95‒2.95)	0.07
TLG (yes vs. no)	27	163	0.04	0.54 (0.30‒0.95)	0.03
Operative time (min), median (IQR)	235 (205‒280)	220 (200‒260)	0.005	1.00 (0.99‒1.01)	0.35
Estimated blood loss (mL), median (IQR)	100 (50‒100)	100 (50‒120)	0.028	1.00 (0.99‒1.01)	0.44
Preoperative Hb (g/L), mean (SD)	125±21	117±23	0.003	0.99 (0.97‒1.01)	0.07
Preoperative NLR, mean (SD)	2.7 ± 1.8	2.5 ± 1.5	0.31		
Preoperative ALB (g/L), mean (SD)	40.3 ± 5.0	38.5 ± 5.0	0.004	0.97 (0.91‒1.04)	0.43
TNM stage (III vs. I & II)	35	104	0.35		
Postoperative complication (yes vs. no)	18	11	<0.001	7.67 (3.24‒18.17)	<0.001

SD, Standard Deviation; IQR, Interquartile; BMI, Body Mass index; TLG, Total Laparoscopic Gastrectomy; Hb, Haemoglobin; NLR, Neutrophil to Lymphocyte Ratio; ALB, Albumin; 95 % CI, 95 % Confidence Interval.

In subgroup analyses comparing TLTG or TLDG with LATG or LADG, patients in the TLTG and TLDG groups exhibited significantly shorter operative times, lesser intraoperative Blood Loss (EBL), and shorter time to first flatus (all *p* < 0.001) (Supplementary Table 2). Notably, the TLTG group demonstrated a lower white blood cell count on POD 1 and a higher serum albumin level on POD 3 compared to the LATG group (*p* < 0.001 and *p* = 0.001, respectively). Interestingly, there were no significant differences in hospital stay duration or medical costs between the TLTG and LATG groups. Conversely, the TLDG group experienced a significantly shorter hospital stay and higher medical costs compared to the LADG group (all *p* < 0.001).

### Postoperative complications

Table 4 shows the incidence of postoperative complications. Specifically, 29 patients (7.6 %) experienced overall postoperative complications, with 4 (1.1 %) classified as major complications. Statistical analysis indicated that the TLG group had a lower incidence of overall complications (9 cases, 4.7 %) compared to the LAG group (20 cases, 10.5 %) (*p* = 0.03). Notably, two patients in the LAG group required re-operation due to anastomotic leakage complicated by severe abdominal infections and active intraperitoneal bleeding, while one patient in the TLG group underwent a re-operation secondary to intraperitoneal bleeding. Importantly, no deaths were observed in either the LAG or TLG groups.

**Table 4:** Postoperative complications.

Variables	TLG group (*n* = 190)	LAG group (*n* = 190)	*p*-value
Postoperative hemorrhage	2	5	0.25
Anastomotic leakage	1	1	1.00
Abdominal abscess	0	1	0.24
Impaired gastric emptying	2	1	0.56
Wound infection	0	4	0.02
DVT	0	1	0.24
Pulmonary infection	4	10	0.10
Major complication	1	3	0.62
Overall complication	9	20	0.03
*Re*-operation	1	2	0.56

DVT, Deep Vein Thrombosis; TLG, Totally Laparoscopic Gastrectomy; LAG, Laparoscopy-Assisted Gastrectomy.

Univariate logistic regression analysis identified several independent predictors of overall complications. TLG was associated with a reduced risk of complications, while prolonged operative time was linked to an increased risk. Further, multivariate logistic regression analysis revealed that a high preoperative ALB level is an independent protective factor for lower overall complication risk (*p* = 0.04), as detailed in Table 5.

**Table 5:** Univariable and multivariable logistic regression analysis for the factors associated with postoperative overall complications.

Variables	Univariate analysis for postoperative complication			Multivariate analysis	
	No (*n* = 351)	Yes (*n* = 29)	*p*-value	Odds Ratio (95 % CI)	*p*-value
Age (years), median (IQR)	63 (54‒69)	64 (55‒73)	0.34		
Sex (female)	100	6	0.37		
BMI (kg/m^2^), median (IQR)	22.5 (20.7‒24.7)	23.8 (20.9‒24.8)	0.28	1.09 (0.95‒1.25)	0.21
ASA (score 3)	210	14	0.22	0.72 (0.33‒1.57)	0.41
Total gastrectomy (yes)	142	10	0.53		
TLG (yes)	181	9	0.03	0.54 (0.23‒1.27)	0.16
Operative time (min), median (IQR)	225 (200‒260)	240 (218‒285)	0.05	1.01 (1.00‒1.02)	0.14
Estimated blood loss (mL), median (IQR)	100 (50‒100)	100 (50‒100)	0.58		
Preoperative Hb (g/L), median (IQR)	126 (111‒139)	127 (114‒138)	0.65		
Preoperative NLR, median (IQR)	2.14 (1.56‒2.97)	1.99 (1.99‒2.41)	0.28	0.73 (0.51‒1.05)	0.09
Preoperative ALB (g/L), median (IQR)	39.9 (36.6‒43.6)	38.8 (33.9‒42.3)	0.21	0.91 (0.84‒0.99)	0.04
TNM stage (III versus I & II)	130	10	0.78		

IQR, Interquartile; BMI, Body Mass Index; TLG, Totally Laparoscopic Gastrectomy; Hb, Haemoglobin; ALB, Albumin; NLR, Neutrophil to Lymphocyte Ratio; ALB, Albumin; 95 %CI, 95 % Confidence Interval.

In subgroup analysis for total or distal gastrectomy, TLTG or TLDG did not increase the risk of overall complications compared to LATG or LADG group, respectively (overall complications, TLTG vs. LATG: 2.6 % vs. 10.5 %, *p* = 0.05; TLDG vs. LADG: 6.2 % vs. 10.5 %, *p* = 0.23) (Supplementary Table 3).

### Oncological outcomes

Table 6 showed the pathological outcomes of the two groups. Compared to LAG group, the TLG group exhibited more lymph node yield (median (IQR): 23 (18‒29) vs. 19 (16‒25), *p* < 0.001). Notably, no significant differences were observed in other key pathologic parameters, including proximal margin, distal margin, tumor diameter, tumor differentiation, and TNM stage between the two groups.

**Table 6:** Pathological outcomes.

Variables	TLG group (*n* = 190)	LAG group (*n* = 190)	*p*-value
Tumor location			0.91
Upper	42	45	
Middle	34	35	
Lower	114	110	
Tumor diameter (cm), median (IQR)	3(2–4.5)	3(2–5)	0.70
Proximal margin (cm), median (IQR)	4(2–6)	4(2–6)	0.14
Distal margin (cm), median (IQR)	3.5(2–6)	3(2–6)	0.06
Total lymph node harvest, median (IQR)	23(18–29)	19(16–25)	<0.001
Positive lymph node harvest, median (IQR)	1(0–4)	1(0–5)	0.88
Histological classification			
Poor	77	64	0.17
Moderate/Well	113	126	
Depth of invasion			0.34
T1	57	58	
T2	43	34	
T3	47	61	
T4	43	37	
N stage			0.85
N0	102	97	
N1	27	30	
N2	35	28	
N3	36	35	
TNM stage			0.19
I	81	73	
II	36	51	
III	73	66	

IQR, Interquartile; TLG, Totally Laparoscopic Gastrectomy; LAG, Laparoscopy-Assisted Gastrectomy.

In subgroup analyses based on surgical approaches, either the TLTG group or TLDG group had more lymph node retrieval compared to the LATG group (median (IQR): 23 (2.018‒29) vs. 20 (16‒24), *p* = 0.012), or LADG group (median (IQR): 23 (18‒29) vs. 19 (15‒25), *p* < 0.001). Additionally, the TLDG group exhibited a longer distal margin (median (IQR): 2.5 (2‒3.5) vs. 2 (1‒3), *p* = 0.002) compared to the LADG group (Supplementary Table 4).

## Discussion

Both LAG and TLG represent minimally invasive approaches for the treatment of gastric cancer. These techniques contribute to reduced surgical trauma, quicker surgical recovery, and shorter hospital stay compared to open surgery.^1^^,^^20^ Although many studies have discussed the feasibility, safety, and efficacy of TLG versus LAG, the outcomes remain inconclusive.^10^, ^11^, ^12^, ^13^^,^^15^^,^^21^^,^^22^ The present study suggests that TLG contributes to a lesser operative time, reduced blood loss, lower risk of postoperative complications, and faster surgical recovery compared to LAG.

First, the present study demonstrated that TLG significantly reduced operative time and intraoperative EBL compared to LAG. Subgroup analyses for total or distal gastrectomy showed consistent results, i.e., reduction in operative time and intraoperative EBL. Additionally, consistent results have been reported in other studies.^13^^,^^20^^,^^23^^,^^24^ Since the surgical procedures of gastric dissection for TLG and LAG are identical, the differences in operative time and EBL are likely attributed to intra- or extracorporeal anastomotic procedures of the digestive tract. In LAG, the anastomotic procedure is performed outside the abdomen with a narrow operative field, which may increase tissue tension and lead to increased blood loss. In contrast, TLG offers a superior surgical field with optimal visualization, minimizing tissue injury and intraoperative bleeding, particularly in obese patients.

Second, the authors observed that patients in the TLG group exhibited faster postoperative recovery, reflected by shorter time to first flatus, shorter time to first liquid diet and shorter length of postoperative stay. Additionally, on postoperative day-3, patients in the TLG group exhibited a higher serum albumin (ALB) level compared to those in the LAG group. The reasons for faster recovery of TLG surgery are probably attributed to: first, TLG reduces abdominal tissue injury and mitigates intestinal disturbance compared to LAG; second, TLG has a shorter operative time and reduces Extracellular Matrix Breakdown (EBL), thereby minimizing surgical trauma and promoting faster recovery. Some studies have reported that TLG operation might reduce postoperative pain, expedite the time to first flatus, accelerate the onset of liquid diet consumption, and shorten the duration of hospitalization after surgery.^13^^,^^24^^,^^25^ However, another study has shown that there is no superiority of TLG in terms of early recovery compared to LAG.^12^^,^^14^^,^^26^ A randomized controlled trial^12^ demonstrated that there were no significant differences in terms of the time to first flatus, the time to first soft diet, and the duration of hospital stay between the two groups.

Third, the present study suggested that TLG contributed to a lower risk of complications compared to LAG, with an overall complication rate of 4.7 % in the TLG group and 10.5 % in the LAG group (*p* = 0.03). Notably, there were no statistically significant differences in major complication risk or reoperation risk between the two groups (*p* = 0.62 for major complications; *p* = 0.56 for reoperation). These results were consistent with results from published studies, ,^12^^,^^14^^,^^24^ including reported by Woo^12^ and Gong.^13^ The randomized controlled trial by Woo^12^ demonstrated that TLDG had a comparable complication risk in comparison to LADG, strengthening the safety and feasibility of TLDG for the treatment of gastric cancer. Furthermore, Gong^13^ reported that the risk of overall complications for TLTG was comparable to that of LATG.

Fourth, the present study showed that TLG had more number of lymph nodes retrieved compared to LAG, which was also reported in some published studies.^9^^,^^14^^,^^25^ In addition, TLDG had a longer distal margin versus LADG (2.5 vs. 2 cm; *p* = 0.002). Interestingly, some studies have reported that TLG has a longer proximal margin than LAG to maintain a negative surgical margin.^8^^,^^13^^,^^24^ A possible reason for explaining the different number of lymph nodes retrieved between the 2 groups was that more patients underwent total laparoscopic gastrectomy more recently, compared to patients undergoing laparoscopy-assisted gastrectomy.

The present study had some limitations. First, the present study was a retrospective study, selection bias of patients might exist.^27^ Second, the sample size for subgroup analysis of total gastrectomy (TLTG vs. LATG) was relatively small. Therefore, a randomized clinical trial with a large sample size is warranted to confirm the findings of the present study.

## Conclusions

Compared to LAG, TLG contributed to lower intraoperative EBL, less surgical trauma, faster surgical recovery and shorter postoperative hospital stay. In addition, TLG had a lower risk of postoperative complications compared to LAG. Thus, these findings suggest that TLG is superior to LAG for the treatment of gastric cancer.

## Data availability

The data files will be available upon request.

## Funding

This research did not receive any specific grant from funding agencies in the public, commercial, or not-for-profit sectors.

## CRediT authorship contribution statement

**Liu Liu:** Conceptualization, Data curation, Methodology, Formal analysis, Writing – review & editing. **Shijie Feng:** Project administration, Formal analysis, Writing – original draft. **Haiyan Wang:** Project administration, Writing – review & editing. **Lin Liu:** Conceptualization, Data curation, Supervision, Validation, Writing – review & editing.

## Declaration of competing interest

The authors declare no conflicts of interest.

## Appendix Supplementary materials

Image, application 1
