# Supplementary material for: Short-term outcome of totally laparoscopic gastrectomy for gastric cancer: a comparative study
Source: Clinics (Sao Paulo). 2026 Feb 19;81:100887. doi: 10.1016/j.clinsp.2026.100887 (PMC12933467; doi:10.1016/j.clinsp.2026.100887)
Supplement: Supplementary file 1 [file mmc1.docx]

**CLINICS-D-25-01767_ Supplementary Materials**

**Supplementary Table 1** Subgroup analysis of baseline characteristics of patients.

|  | **Total gastrectomy** | | | **Distal gastrectomy** | | |
| --- | --- | --- | --- | --- | --- | --- |
| **Variables** | **TLTG group**  **(n = 76)** | **LATG group**  **(n = 76)** | **p-value** | **TLDG group**  **(n = 114)** | **LADG group**  **(n = 114)** | **p-value** |
| Age (years), (mean ± SD) | 65.0 ± 10.7 | 66.0 ± 10.4 | 0.60 | 60.0 ± 10.2 | 60.1 ± 10.5 | 0.89 |
| Sex |  |  | 0.67 |  |  | 0.95 |
| Male | 59 | 55 |  | 79 | 80 |  |
| Female | 18 | 21 |  | 33 | 34 |  |
| BMI (kg/m^2^), (mean ± SD) | 22.2 ± 2.7 | 22.8 ± 2.9 | 0.38 | 23.3 ± 2.9 | 22.4 ± 2.9 | 0.03 |
| Charlson Comorbidity Index (CCI) |  |  | 0.55 |  |  | 0.47 |
| 0‒1 | 71 | 69 |  | 106 | 103 |  |
| > 1 | 5 | 7 |  | 8 | 11 |  |
| ASA score |  |  | 0.26 |  |  | 0.31 |
| I & II | 34 | 41 |  | 30 | 37 |  |
| III | 42 | 35 |  | 84 | 77 |  |
| History of abdominal surgery |  |  | 0.12 |  |  | 0.70 |
| Yes | 3 | 8 |  | 15 | 17 |  |
| No | 73 | 68 |  | 99 | 97 |  |
| Preoperative WBC (×10^9^/L), (mean ± SD) | 5.8 ± 1.6 | 5.6 ± 1.4 | 0.29 | 5.9 ± 1.5 | 5.7 ± 1.8 | 0.28 |
| Preoperative NLR, (mean ± SD) | 2.2 ± 1.7 | 2.2 ± 1.5 | 0.27 | 2.6 ± 1.6 | 2.3 ± 1.4 | 0.15 |
| Preoperative Hb (g/L), (mean ± SD) | 123 ± 25 | 119 ± 21 | 0.28 | 127 ± 21 | 126 ± 19 | 0.78 |
| Preoperative ALB (g/L), (mean ± SD) | 40.8 ± 5.1 | 38.1 ± 5.6 | 0.49 | 40.4 ± 4.9 | 40.4 ± 4.6 | 0.97 |

SD, Standard Deviation; BMI, Body Mass Index; WBC, White Blood Cell; NLR, Neutrophil to Lymphocyte Ratio; Hb, Haemoglobin; ALB, Albumin; TLTG, Totally Laparoscopic Total Gastrectomy; LATG, Laparoscopy-Assisted Total Gastrectomy; TLDG, Totally Laparoscopic Distal Gastrectomy; LADG, Laparoscopy-Assisted Distal Gastrectomy.

**Supplementary Table 2** Subgroup analysis of surgical outcomes and postoperative recovery.

|  | **Total gastrectomy** | | | **Distal gastrectomy** | | |
| --- | --- | --- | --- | --- | --- | --- |
| **Variables** | **TLTG group (n = 76)** | **LATG group (n = 76)** | **p-value** | **TLDG group (n = 114)** | **LADG group (n = 114)** | **p-value** |
| Operative time (min), median (IQR) | 235 (200‒277) | 260 (225‒300) | 0.005 | 210 (189‒236) | 230 (200‒261) | 0.001 |
| Estimated blood loss (mL), median (IQR) | 90 (50‒100) | 100 (100‒150) | <0.001 | 50 (50‒100) | 100 (50‒200) | <0.001 |
| First flatus (days), median (IQR) | 3.9 (3.3‒4.6) | 4 (4‒5) | 0.004 | 3 (3‒3) | 4 (4‒5) | <0.001 |
| Liquid diet (days), median (IQR) | 4 (4‒4) | 6 (5‒6) | <0.001 | 3 (3‒4) | 5 (4‒6) | <0.001 |
| Postoperative hospital stay (days), median (IQR) | 8 (7‒9) | 9 (8‒10) | 0.13 | 7 (7‒8) | 8 (7‒9) | <0.001 |
| WBC (×10^^9^/L), mean (SD) |  |  |  |  |  |  |
| POD1 | 11.33 ± 3.10 | 13.05 ± 3.48 | <0.001 | 12.00 ± 3.08 | 12.54 ± 3.60 | 0.23 |
| POD3 | 8.07 ± 2.14 | 7.34 ± 2.85 | 0.03 | 8.00 ± 2.42 | 7.97 ± 2.42 | 0.94 |
| NLR, mean (SD) |  |  |  |  |  |  |
| POD1 | 15.95 ± 22.41 | 18.09 ± 13.09 | 0.44 | 14.63 ± 13.17 | 18.45 ± 19.46 | 0.08 |
| POD3 | 8.05 ± 7.88 | 7.23 ± 6.20 | 0.43 | 6.93 ± 4.69 | 8.51 ± 6.70 | 0.04 |
| ALB (g/L), mean (SD) |  |  |  |  |  |  |
| POD1 | 34.3 ± 3.69 | 33.26 ± 4.63 | 0.07 | 35.72 ± 3.95 | 35.28 ± 3.95 | 0.40 |
| POD3 | 34.86 ± 4.37 | 32.79 ± 3.58 | 0.001 | 36.04 ± 4.06 | 35.94 ± 4.84 | 0.87 |
| Medical cost (CNY), median (IQR) | 49229 (30696‒107079) | 46770 (35700‒78694) | 0.19 | 56744 (52454‒62612) | 44232 (52454‒62612) | <0.001 |

IQR, Interquartile; SD, Standard Deviation; WBC, White Blood Cell; NLR, Neutrophil to Lymphocyte Ratio; ALB, Albumin; TLTG, Totally Laparoscopic Total Gastrectomy; LATG, Laparoscopy-Assisted Total Gastrectomy; TLDG, Totally Laparoscopic Distal Gastrectomy; LADG, Laparoscopy-Assisted Distal Gastrectomy.

**Supplementary Table 3** Subgroup analysis of postoperative complications after surgery.

|  | **Total gastrectomy** | | | **Distal gastrectomy** | | |
| --- | --- | --- | --- | --- | --- | --- |
| **Variable** | **TLTG group**  **(n = 76)** | **LATG group**  **(n = 76)** | **p-value** | **TLDG group (n = 114)** | **LADG group (n = 114)** | **p-value** |
| Postoperative hemorrhage | 0 | 2 | 0.16 | 2 | 3 | 0.65 |
| Anastomotic leakage | 1 | 1 | 1.00 | 0 | 0 | NA |
| Abdominal abscess | 0 | 1 | 0.24 | 0 | 0 | NA |
| Impaired gastric emptying | ‒ | ‒ | NA | 2 | 1 | 0.56 |
| Wound infection | 0 | 2 | 0.09 | 0 | 2 | 0.10 |
| DVT | 0 | 0 | NA | 0 | 1 | 0.24 |
| Pulmonary infection | 1 | 4 | 0.18 | 3 | 6 | 0.51 |
| Major complication | 0 | 1 | 1.00 | 1 | 2 | 1.00 |
| Overall complication | 2 | 8 | 0.05 | 7 | 12 | 0.23 |
| Re-operation | 0 | 1 | 0.24 | 1 | 1 | 1.00 |

DVT, Deep Vein Thrombosis; TLTG, Totally Laparoscopic Total Gastrectomy; LATG, Laparoscopy-Assisted Total Gastrectomy; TLDG, Totally Laparoscopic Distal Gastrectomy; LADG, Laparoscopy-Assisted Distal Gastrectomy; NA, Not Applicable.

**Supplementary Table 4** Subgroup analysis of pathological outcomes.

|  | **Total gastrectomy** | | | **Distal gastrectomy** | | |
| --- | --- | --- | --- | --- | --- | --- |
|  | **TLTG group**  **(n = 76)** | **LATG group**  **(n = 76)** | **p-value** | **TLDG group**  **(n = 114)** | **LADG group**  **(n = 114)** | **p-value** |
| **Tumor location** |  |  | 0.37 |  |  | 0.16 |
| Upper | 42 | 45 |  | 0 | 0 |  |
| Middle | 24 | 17 |  | 10 | 18 |  |
| Lower | 10 | 14 |  | 104 | 96 |  |
| Tumor diameter (cm), median (IQR) | 3.5 (2.0‒5.0) | 4.0 (2.5‒5.4) | 0.21 | 3.0 (2.0‒4.0) | 2.7 (1.5‒4.4) | 0.46 |
| Proximal margin (cm), median (IQR) | 1.8 (1.0‒3.0) | 1.5 (1.0‒2.0) | 0.09 | 5 (4‒7) | 5 (4‒6) | 0.33 |
| Distal margin (cm), median (IQR) | 7.3 (5.0‒9.9) | 7.0 (5.0‒9.0) | 0.66 | 2.5 (2‒3.5) | 2 (1‒3) | 0.002 |
| Total lymph node harvest, m median (IQR) | 23 (18‒29) | 20 (16‒24) | 0.012 | 23 (18‒29) | 19 (15‒25) | <0.001 |
| Positive lymph node harvest, median (IQR) | 1 (0‒4) | 2 (0‒7) | 0.34 | 1 (0‒4) | 0 (0‒4) | 0.29 |
| **Histological classification** |  |  | 0.22 |  |  | 0.42 |
| Poor | 27 | 20 |  | 50 | 44 |  |
| Moderate/well | 49 | 56 |  | 64 | 70 |  |
| **Depth of invasion** |  |  | 0.68 |  |  | 0.25 |
| T1 | 15 | 12 |  | 42 | 46 |  |
| T2 | 15 | 11 |  | 28 | 23 |  |
| T3 | 23 | 28 |  | 24 | 33 |  |
| T4 | 23 | 25 |  | 20 | 12 |  |
| **N stage** |  |  | 0.45 |  |  | 0.40 |
| N0 | 46 | 32 |  | 56 | 65 |  |
| N1 | 11 | 11 |  | 16 | 19 |  |
| N2 | 14 | 13 |  | 21 | 15 |  |
| N3 | 15 | 20 |  | 21 | 15 |  |
| **TNM stage** |  |  |  |  |  | 0.65 |
| I | 27 | 19 | 0.12 | 54 | 54 |  |
| II | 13 | 23 |  | 23 | 28 |  |
| III | 36 | 34 |  | 37 | 32 |  |

TLTG, Totally Laparoscopic Total Gastrectomy; LATG, Laparoscopy-Assisted Total Gastrectomy; TLDG, Totally Laparoscopic Distal Gastrectomy; LADG, Laparoscopy-Assisted Distal Gastrectomy.
